# Sorting it out: perceptions of foods among newly arrived adolescent refugees in the Southeastern USA

**DOI:** 10.1017/S1368980024002544

**Published:** 2024-12-26

**Authors:** Rebecca E Jones-Antwi, Caroline Owens, Craig Hadley, Solveig A Cunningham

**Affiliations:** 1 Department of Public Health, Baylor University, Waco, TX 76798, USA; 2 Department of Epidemiology, Emory University, Atlanta, GA 30322, USA; 3 Hubert Department of Global Health, Emory University, Atlanta, GA 30322, USA; 4 Department of QTM and Anthropology, Emory University, Atlanta, GA 30322, USA; 5 Gerald J. and Dorothy R. Friedman School of Nutrition Science and Policy, Tufts University, Boston, MA 02111, USA

**Keywords:** Food, Diet, Acculturation, Cultural consensus analysis, Adolescents, Refugees

## Abstract

**Objective:**

To explore the meanings that newly arrived refugee adolescents residing in the Southeastern USA attribute to foods.

**Design:**

We used methods from cognitive anthropology to assess whether adolescents from different countries share a cultural model of eating behaviours.

**Setting:**

A school-based study in a community in the Southeastern USA.

**Participants:**

Adolescents (10–17 years) who arrived in the USA on a refugee visa in the previous year.

**Results:**

Adolescents showed consensus in grouping items and in identifying some foods as associated with adults and others with children. There was evidence of a shared model of eating practices across age, gender and number of siblings. Adolescents who had lived in a refugee camp were significantly different in how they grouped items.

**Conclusions:**

Adolescents from nine countries shared a model of eating behaviours; these patterns are consistent with rapid dietary acculturation within 1 year of arrival or with shared models held from pre-arrival. Our finding that adolescents who recently arrived in the USA generally agree about how foods relate to one another holds promise for generalised nutrition and dietary interventions across diverse adolescent groups.

Many people resettling in a new country experience shifts in norms and lifestyles that may affect their dietary and activity habits and preferences^(1–6)^. Importantly, the speed and type of lifestyle change may not be uniform across immigrants. For example, immigrant children often experience more rapid linguistic adaptation and greater exposure to American culture than immigrant adults^(7,8)^. Child immigrants entering US society and schools during sensitive ages may be particularly susceptible to adopting unhealthy dietary behaviours common in the USA. Supporting this hypothesis, Van Hook and colleagues (2018) found that Mexican immigrants who arrived during preschool and school ages have less healthy diets than Mexican immigrants who arrived as adults^(9)^. Given the relevance of diet in shaping life course well-being, the processes through which dietary and lifestyle changes manifest are of scientific and programmatic importance.

Eating is one of the most profoundly meaning-ridden activities in human life^(10–13)^. Food habits reproduce class distinctions and reinforce membership across ethnic, religious, gendered and social categories^(12,14,15)^. Thus, while factors such as availability and accessibility make certain food choices convenient, socio-culturally mediated meanings are implicit in every food choice an individual makes. In other words, ‘food is never just food’^(15)^. In an exploration of this phenomenon among adolescents in India, Maxfield and colleagues found that adolescents generally agreed that non-local foods and foods consumed outside the home were seen as most prestigious. Their analysis demonstrates that changes in local foodways impact beliefs about the status and meaning conveyed by foods and eating among young people^(10)^. Related work with adolescents in India demonstrated nuance in selection of foods^(16)^; for example, food selection was influenced by price for some food groups but not others (e.g. snacks *v*. fruits). Food decisions are complex and may be guided by external cues, including dietary recommendations, marketing, price, ease of preparation, packaging and product placement, as well as internal cues, such as taste preference for sugar and fat^(17,18)^. In immigrant communities, children may strive to meet ideals of normative eating shared by classmates and peers. These normative ideals around eating may not be shared by their adult family members. Harris, Mullan and Chen refer to this intergenerational dissonance as an ‘acculturation gap’, finding that second-generation adolescents share weekly dinners with parents less frequently and encounter more serious arguments with parents about behaviours than their first-generation counterparts^(19,20)^. Sociologists and other social scientists have richly documented the complex negotiations parents in general, and immigrant parents in particular, must make to feed their families.

Food choices are influenced by family and social contexts^(21)^. Research in the USA demonstrates that parent and child food preferences align in some areas but are often not identical^(22)^. Previous studies have demonstrated differentiation in parent–child diets as children reach adolescence^(23)^. A systematic review of nutrient intake between parents and children living in the USA concluded there was only a weak resemblance in nutrient intake^(24)^. Another systematic review and meta-analysis of parent–child dietary resemblance utilising more comprehensive measures of diet found weak to moderate resemblance of dietary intake, indicating that parents’ dietary intake behaviour does not fully shape child dietary intake^(25)^. Parents in general, and immigrant parents in particular, engage in complex negotiations to feed their families. Beliefs immigrant adolescents associate with different foods advances our understanding of nutrition and food choice. Therefore, examining the values and beliefs immigrant adolescents associate with different foods when categorising foods into parent, child and joint consumption groups advances our understanding of nutrition and food choice.

The aim of this study was to assess whether young people from different countries who migrated to the USA on a refugee visa agree on the meaning and values of different foods – that is, do they share ideas about categories of foods. With respect to foods and beverages they no longer consumed and those they newly adopted after migration, we examine how these are categorised in the eyes of these young immigrants, and what foods/beverages are considered to be ‘adult foods’ *v*. ‘kids’ foods’.

Our study was based in an afterschool programme, and all study participants attended this programme and lived in the same community. Given that these adolescents are immersed in a shared environment at school and in their community of resettlement, we hypothesise that they will exhibit shared cultural models of food, eating practices and social meaning *despite* their diverse backgrounds and experiences.

## Methods

### Setting and sample

Data collection was conducted in a community in the Southeastern United States noted for its ethnic diversity, often referred to as the ‘most diverse square mile in America’ and ‘the Ellis Island of the South’^(26)^. Since 1980, more than 60 000 refugees have relocated to the town and its surrounding area. The first wave of refugees to resettle in the area were refugees from Vietnam and other Southeast Asian countries. Refugee resettlement to the community increased significantly in the 1990s – the community’s foreign-born population was 9 % in 1990 and doubled by 1995. This community became a prime resettlement location based on the surrounding city’s robust economic growth, while the smaller community has affordable housing options, access to public transportation and proximity to downtown Atlanta. By 2023, nearly half of residents are foreign-born, with more than 150 ethnic groups and sixty languages spoken^(27)^. This demographic context may entail specific dietary practices among adolescents because, unlike many cities across the USA, diverse food environments are easily accessible within a 1-mile radius.

This study was part of a larger project focused on identifying ways in which refugees assimilate into their new communities and how assimilation may affect their risk of obesity and diabetes. We partnered with a refugee resettlement organisation that runs an afterschool programme providing social and academic support to refugee youth as they adapt to life in the USA. During each academic year, four cohorts of 20–30 students participated in the programme. The programme is for students 10–17 years of age within 1 year of arrival and is delivered over 6 weeks. We conducted data collection from November 2017 to May 2018 with three cohorts of the after-school programme. All seventy-five adolescents enrolled in the programme were invited to participate in this study, and all agreed to participate. Due to migration away from the area and incomplete attendance by the students in this optional programme, we have complete data for sixty-eight students and partial data for seven more.

Characteristics of the newly arrived refugee adolescents participating in the after-school programme are shown in Table 1. Adolescents were from nine countries of origin. Thirty-seven (49 %) had spent some time in a refugee camp prior to migration to the USA. Fifty (66·7 %) were from either Afghanistan or the Democratic Republic of Congo. The average time since arrival was 5·4 months, with 1 year being the longest an adolescent in the programme had lived in the USA. We did not collect data on religion or ethnicity at to our community partner’s request.

**Table 1.** Characteristics of adolescent participants of afterschool programme run by resettlement organisation

	Cohorts 2–4 (*n* 75)	
Variable	Mean or *number*	sd or/%
Age (years)	14·4	2·20
Female	39	52 %
Length of stay (months)	5·4	5·84
Country of origin		
Afghanistan	25	33 %
Bhutan	3	4 %
Burma	9	12 %
Democratic Republic of the Congo	25	33 %
Ethiopia	2	2·7 %
Eritrea	3	4 %
Somalia	5	6·7 %
Syria	2	2·7 %
Sudan	1	1·3 %
Native language		
Arabic	3	4 %
Burmese	4	5·3 %
Dari	22	29 %
Farsi	3	4 %
Karen	5	6·7 %
Kinyarwanda	4	5·3 %
Nepali	3	4 %
Somali	5	6·7 %
Swahili	22	29 %
Tigrigna	4	5·3 %
Number of siblings	3·9	2·07
Single mother	7	9·3 %
Currently lives with parents	70	93 %
Lived in a refugee camp (yes)	37	49 %
Country of refugee camp^*^		
Ethiopia	2	5·4 %
Kenya	1	2·7 %
Namibia	2	5·4 %
Nepal	3	8·1 %
Rwanda	4	10·8 %
Tanzania	16	43·2 %
Thailand	5	13·5 %
Uganda	4	10·8 %

* Proportion calculated from those who had lived in a refugee camp (*n* 37).

### Data and methods

We rely on methods from cognitive anthropology to generate food and beverage lists (i.e. freelisting) and to determine whether youths grouped these foods into similar clusters (i.e. pilesorting)^(28)^. We postulate that young people who categorise food and beverages in a similar fashion may be drawing on a shared cultural model, or belief set, when deciding how to categorise items. As defined by Cultural Model Theory, culture refers to mental knowledge shared by a social group that may or may not be territorially defined; expanding this idea, cultural models refer to constitutive knowledge or belief sets related to a theme^(29)^. If adolescents’ cultural models differ by gender or country of origin or other characteristics, then resettlement programming around health and nutrition will need to be tailored for these groups. From an empirical lens, investigating the cultural models that adolescents hold around food and eating behaviours may shed light on the social mechanisms of dietary change and related health outcomes after migration.

As outlined in detail below, we conducted structured freelist interviews with a subsample of adolescents to generate a list of foods and beverages that they consumed before and after moving to the USA (*n*=26). Freelist interviews identified culturally relevant foods and beverages to include in our analysis. Preserving the thirty-two most frequently listed items, we generated a set of picture cards, conducted a pilesort activity with a different group of adolescents (*n* 68) and interviewed them about how they sorted the cards. Through the pilesort technique, we aim to assess whether adolescents agree about *who* eats certain types of foods and beverages. This is a step towards an exploration of how adolescents sort between parent and child-specific food and beverage items – a topic to be studied in future analyses. Finally, we conducted cultural consensus and regression analyses to examine factors that inform differences in how adolescents conceptualise and categorise food and beverage items.

### Instrument development

With guidance and feedback from our community partner, we developed freelist and pilesort instruments (described below) to evaluate lifestyle changes. In the summer of 2017, we met with caseworkers, nutrition educators and other staff at the resettlement organisation to determine key aspects they wanted to glean from the project. We adapted a freelist instrument from our previous work with refugee adults at the point of green card status in the same city at one refugee resettlement office 2016 through 2017^(30)^. In adapting instruments, we conducted cognitive interviews with three immigrant adolescents of the same age range as adolescents enrolled in the afterschool programme. With the first cohort of students (*n* 26), we evaluated English proficiency and establish how to integrate research into the programme (pre-test cohort). We pre-tested the revised instruments again with three new students. We created a demographic intake sheet with information about age (years), date of arrival in the USA, country of origin, household composition and whether they had ever lived in a refugee camp. The youth programme supervisor filled this form out for every student after consent from parents was received. All instruments were developed in English and were administered in the language of each adolescent’s choice by an interpreter. We were not able to translate instruments in advance, as the programme coordinators did not know in advance which youths would be arriving and consequently which languages would be spoken; therefore, we used our community partner’s interpreters who were already at the school for the programme. A study team interviewer worked with each interpreter and recroded. The answers in English based on the interpreter’s translation.

### Freelisting

Freelisting is a semi-structured interview method that asks participants to name all the items they can think of for a particular domain; in this study, the domain of interest was food and beverages consumed^(28)^. Generally, respondents list first the items most familiar, or salient, to them. Items that are frequently listed across individuals tend to appear early in individual lists. We developed a freelist questionnaire that asked participants to list three food and beverage items for two prompts: (1) ‘What are things you eat or drink very often now that you rarely ate or drank before you came to the United States?’ and (2) ‘Tell me things that you ate or drank most frequently before coming to the United States that you rarely eat now’. These prompts were asked among the pre-test cohort of the programme (*n*=26; cohort 1). From the thirty-two most frequently listed foods and beverages generated through the freelists (further delineated in Table 2), we created a set of picture cards for pilesort exercises (Table 3). The final picture cards of foods and beverages were also informed by trips to grocery stores in the area, previous data from adult refugees in the same community^(28)^, discussions with the nutrition educator at the resettlement organisation, and caseworkers at two other resettlement organisations in the area. The thirty-two foods and beverages picture cards were placed on laminated cards for data collection (see Appendix Fig. A.1 for pictures of all cards).

**Table 2.** Items adolescents listed as ‘Foods They Stopped Eating’ and ‘Foods They Started Eating’ after migration (*n* 26)

Items stopped eating or drinking			Items started eating or drinking		
	%	57·6 %		%	61·0 %
	Mean # (Range)	2·8 (1–4)		Mean # (Range)	2·9 (1–7)
Rank	Food/Drink	Frequency	Rank	Food/Drink	Frequency
1	**Rice**	6	1	**Pizza**	8
2	**Beans**	5	2	Chicken	6
3	**Potato**	4	3	Apple	5
3	Chicken	3	4	Pig	4
3	**Milk**	3	5	**Fish**	3
3	**Pizza**	3	5	Oranges	3
7	**Vegetables**	2	5	**Juice**	3
7	**Fish**	2	5	**Chips**	3
9	**Fruits**	1	9	**Beans**	2
9	Tomatoes	1	9	Grapes	2
9	Bamboo	1	11	**Pasta**	1
9	**Juice**	1	11	**Rice**	1
9	Ugali	1	11	**Coke**	1
9	Pig	1	11	**Meat**	1
9	Sweet Potato	1	11	Lentils	1
9	**Meat**	1	11	**Bread**	1
9	**Chocolate Milk**	1	11	**Chicken Nuggets**	1
9	Cassava Leaves	1	11	Strawberries	1
9	**Mango**	1			
9	Chili	1			
9	**Soda**	1			
9	Fish Paste	1			

All foods and beverages listed are included.

Bolded items were used in pilesort and consensus surveys.

**Table 3.** Items identified in rreelist, which were selected for use as cards during pilesort

Card item
1. Pizza
2. Juice (4 flavours)
3. Mixed Nuts
4. Pasta
5. Roasted Potatoes (homemade)
6. McDonalds French Fries
7. Cocacola
8. Black Beans (homemade)
9. Baked Salmon (homemade)
10. Kale salad (homemade)
11. Buffy’s Cool Cow Chocolate Milk (5 % Low Fat)
12. Whole Milk
13. Mango
14. Rye Bread
15. Avocado
16. Candy bars (picture includes multiple kinds)
17. Cheeseburger
18. Goldfish snack packet (Pepperidge Farm)
19. Ground Beef Tacos (homemade)
20. Sargento String Cheese (Part Skim Mozzarella)
21. Chips (picture includes multiple kinds)
22. Cheese (picture includes multiple kinds)
23. Kiwi
24. OnCor Breaded Chicken Parmagiana Patties
25. Vlasic Original Dill Pickles Whole
26. Lunchables Turkey and Cheddar with Crackers
27. Flavoured Yogurt Drinks
28. GoGoSqueexe applesauce (apple cinnamon)
29. Quaker Popped Rice Crisps (BBQ)
30. Fibre One Baked Bars (Lemon)
31. Cheerios
32. White Rice (homemade)

### Pilesorting

In the pilesorting method, participants sort cards created from the freelist into categories based on their own perceptions and preferences. Pilesorts allow researchers to identify which items people think relate to each other; this method gives insights into participants’ conceptual models, in this case those related to food^(28)^. To initiate an unconstrained pilesort, the interviewer asked each adolescent to arrange the shuffled deck of thirty-two cards into categories however he or she wanted. This method was used to identify differences in food groupings. Adolescents were required to make at least two piles, each pile containing at least two cards, but no limit was placed on the number of piles. Interviewers emphasised there were no correct answers. After the cards were sorted into categories, interviewers asked adolescents to explain the piles created. The interviewers elicited names for each pile with pre-scripted questions ‘Why do these foods and drinks belong together?’; responses were recorded verbatim as pile names.

The adolescents then completed a second pilesort exercise called a constrained pilesort pertaining to perceived generational differences in foods. The interviewer asked each participant to organise the deck of thirty cards into a pile for ‘what parents eat or drink’, a pile for ‘what kids eat or drink’ and a pile for ‘what both parents and kids eat or drink’; if a card did not belong, it would be placed in a fourth ‘don’t belong’ pile. After the cards were sorted into groups, interviewers asked participants to explain why cards were placed into the respective piles and verbatim recorded participants’ responses.

### Analytic methods

To analyse freelist data, we calculated the frequency with which adolescents listed each item. Some of the items were collapsed into broader, culturally relevant items^(28)^. For example, brand names such as ‘Coca-Cola’ were collapsed into the item ‘Soda’. The decision to collapse an item was informed by discussions with caseworkers, nutrition educators and other staff at the resettlement organisation and the responses of participants.

To analyse pilesort data, we used cultural consensus analysis, exploratory multidimensional scaling (MDS) and regression analyses. The pilesorts were analysed with Visual Anthropac^(31)^. As part of exploratory data analysis, MDS plots illustrate spatial patterns of proximity in a pilesort task – that is, they provide a graphic depiction of the aggregate grouping of items. To derive coordinates for the MDS plot, item-by-item aggregate proximity matrices were generated. Stress level, which is a goodness-of-fit measure, was calculated for all MDS plots. Larger stress levels indicate poorer fit. For a thirty-item matrix scaled in two dimensions, a stress level below 0·3 is considered acceptable and suggests only a 1 % chance of items in the MDS plot being randomly arranged^(32)^. Using Anthropac, we performed hierarchical clustering analysis on food items to assess grouping within the domains. Clusters are partitioned using the aggregate proximity matrix data. The clustered terms signify different domains of thought related to the sorting exercise. To determine the optimal number of clusters to reach cluster homogeneity and to interpret cluster meaning, we compared clustering at different partition levels^(33)^. We assigned clusters numbers to assist with interpretation. The ordinal position of clusters relative to one another is arbitrary. For enhanced visualisation, the food item coordinate data and clusters calculated in Visual Anthropac were exported to R (R Core Team, 2014). Using coordinate data, plots were produced in R using scatterplot functionality available with the ggplot2 package^(34)^.

We adopted principles from cognitive anthropology to calculate cultural competence measures using cultural consensus analysis for both constrained and unconstrained pilesorts^(28)^. Cultural consensus analysis measures the reliability of participant responses in relation to one another and the overall group. Through this process, participants are assigned measures for how accurately their given domain corresponds to the domain in the group. Therefore, cultural competence measures indicate how close individual participants were to the cultural domain of the group. The higher the competence score, the more an individual’s piles matched the cultural consensus of the whole group. For cultural consensus analysis, an eigenvalue ratio of at least 3:1 is considered evidence of a single, shared cultural model; a ratio of 4:1 is sometimes used as a more stringent cut-off^(35)^. Linear regression models were run with competence as the outcome and covariates being age (continuous), gender (male, female), region (Africa (Ethiopia, Eritrea, Somalia, Sudan, Democratic Republic of the Congo (DRC)), Middle East (Afghanistan, Syria), Asia (Bhutan, Burma)), number of siblings (continuous) and lived in a refugee camp prior to US arrival (yes, no). STATA 17.0 was used for regression analyses. We ran a separate model for constrained and unconstrained pilesort competence measures. We do not include length of time in the USA in models because all respondents were within 1 year of arrival.

Several participants were from the same household, for example, siblings or cousins living together (thirty-eight households total); therefore, as a sensitivity analyses, we re-estimated the MDS plots and competence measures with only one adolescent per household selected randomly.

## Results

### Foods refugee adolescents stopped and started eating after migration

Over half of adolescents (*n* 26) reported no longer having certain foods and drinks they used to have before migration to the USA (58 %) (Table 2). They listed an average of 2·8 foods and drinks they had stopped eating, with a range of 1–5 items listed. Rice, beans and potatoes were the top three items listed as no longer eaten. A larger number of adolescents reported adopting new foods and drinks since living in the USA (61 %), listing on average 2·9 foods and drinks they had started consuming, with a range of 1–7 items listed. Pizza, chicken and apple were the top three items listed as newly adopted.

### Refugee adolescents exhibit consensus about food groups

**Figure 1.**
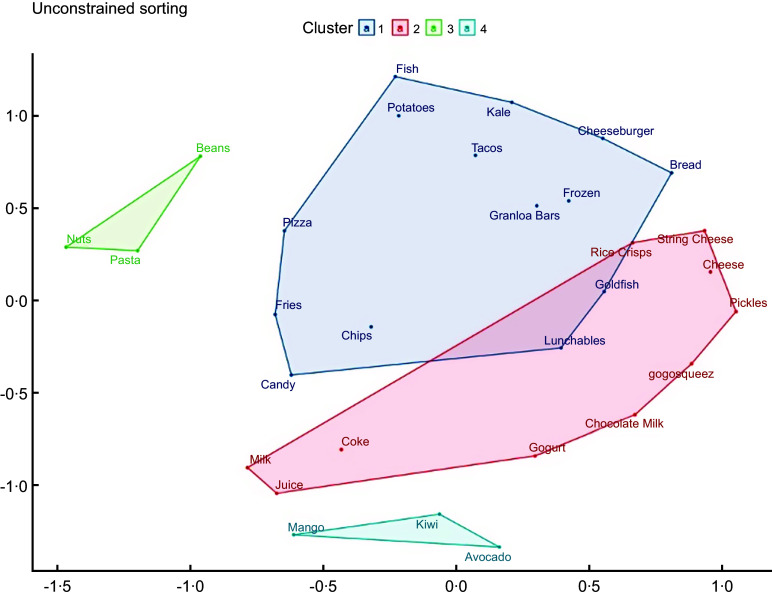
Multidimensional scaling plot (stress = 0·221) and cluster analysis for adolescents’ (*n* 68) unconstrained pilesorts of thirty foods and beverages. *Note:* 1: American Foods; 2: Snacks and Drinks; 3: Dense Foods; 4: Fruits.

Items grouping created by the adolescents yielded four themes, qualitatively described by the adolescents as ‘American foods’, ‘Snacks and Drinks’, ‘Dense or Heavy Foods’ and ‘Fruits’ (Fig. 1). Cluster 1 (American Foods) consisted of fourteen food items: pizza, fries, chips, candy, fish, roasted potatoes, kale, tacos, cheeseburger, frozen fish, granola bars, bread, goldfish and Lunchables. This cluster has some overlap with cluster 2 due to a lack of consensus in the placement of goldfish and Lunchables. Cluster 2 (Snacks and Drinks) consisted of ten items: rice crisps, string cheese, regular cheese, pickles, Gogosqueez apple sauce, chocolate milk, Gogurt, Coke, juice and milk. The proximity of beverages to one another even within the cluster highlights that beverages were frequently grouped together. Both cluster 1 and 2 spatially took up a lot of the axes, indicating not as much consensus as clusters 3 and 4. Cluster 3 (Dense or Heavy Foods) consisted of three items: pasta, nuts and black beans. Cluster 4 (Fruits) consisted of three items: mango, kiwi and avocado. Items in Cluster 3 and 4 were consistently grouped together, and consensus was high. The MDS stress level suggests this domain structure was not random (stress = 0·221), and the eigenvalue ratio was 3·26, indicating that young people shared their understanding of how these foods ‘should’ be sorted. In robustness checks, when evaluating only one adolescent per household (Appendix Fig. A.2), four similar groups still emerged; the MDS stress level and eigenvalue ratio also still suggested that this domain structure is not random (stress = 0·211; eigenvalue ratio: 5·31).

### Constrained pilesort- ‘American Foods’ identified for both parents and adolescents

**Figure 2.**
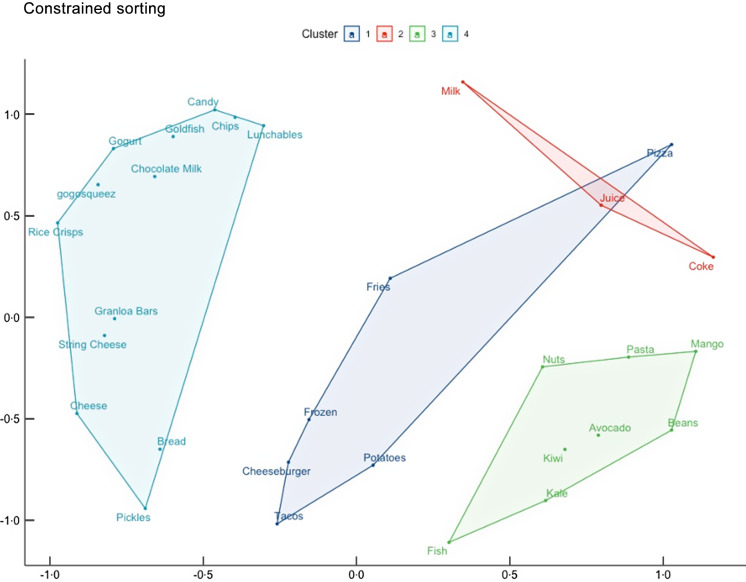
Multidimensional scaling plot (stress = 0·190) and cluster analysis for adolescents’ (*n* 68) constrained pilesorts of thirty foods and beverages. *Note:* 1: Both; 2: Don’t Belong; 3: Parent Foods and Drinks; 4: Adolescent Foods and Drinks.

When subsequently prompted to sort the picture cards into item for, kids, or both, the adolescents’ grouping yielded four clusters (Fig. 2). Cluster 1 (both Parents and Adolescents) consisted of six items: tacos, cheeseburger, roasted potatoes, French fries and pizza. This cluster had some overlap with cluster 2, which was a catchall for items the adolescents felt did not belong in any group (Don’t Belong) and which consisted of beverages: coke, juice and milk, perhaps suggesting that the youth did not conceptualise of beverages in terms of generational consumption. Cluster 3 – Parent Foods – consisted of eight items: mango, pasta, black beans, avocado, nuts, kiwi, kale and fish. Cluster 4 –Kids Stuff – had thirteen items: candy, chips, Lunchables, chocolate milk, Gogurt, GogoSqueez, rice crisps, granola bars, string cheese, cheese, bread and pickles. Cluster 3 and 4 were spatially the furthest apart from one another, with Cluster 1 being in the middle, indicating that parent and kid food and drink designations are distinct and items for both groups are clustered among them. The MDS stress level suggests this domain structure was not random (stress = 0·190), and the eigenvalue ratio was 6·08, indicating that young people shared in their understanding of how these foods relate to one another.

When evaluating only one adolescent per household (Appendix Fig. A.3), the groups were slightly different but consistent spatially; the MDS stress level and eigenvalue ratio also still indicated that this domain structure is not random (stress = 0·224; eigenvalue ratio: 5·25).

These results indicate that, in general, adolescents within 1 year of arrival to the USA agree on which foods are conceived of being related.

### Food consensus and refugee camp experience

We fit two regression models to assess what characteristics of participants were most associated with competence, that is, with sharing the overall group mental model (Table 4). When evaluating individual agreement with the unconstrained groupings of food and beverages (i.e. general differences in food groupings), there were no differences in how adolescents grouped the cards by gender, age, number of siblings or region of origin. Those who had lived in a camp prior to arrival exhibited more competence or agreement, grouping items more like the answers of the overall group compared with those who did not live in a camp (β (95 % CI); 0·22 (0·02, 0·43)). However, in the constrained groupings focused on generational allocation of items, adolescents from countries in the Middle Eastern region exhibited less agreement in grouping items for adults and children more similarly to the cultural consensus answers compared with adolescents from countries in Africa (–0·24 (–0·42, –0·04)).

**Table 4.** The reliability of responses in pilesorts in relation to one another and the overall group using competence scores as outcome with demographics as predictors

	Full sample (*n* 68)				One adolescent per household (*n* 38)			
	Generational differences^a^		General piles^b^		Generational differences^a^		General piles^b^	
	β	95 % CI	β	95 % CI	β	95 % CI	β	95 % CI
Gender (Ref: Male)								
Female	0·09	–0·01, 0·20	0·01	–0·12, 0·14	0·03	–0·11, 0·17	0·02	–0·16, 0·20
Age	−0·0006	–0·03, 0·03	0·003	–0·03, 0·03	0·02	–0·02, 0·05	0·03	–0·01, 0·07
Number of siblings	−0·0004	–0·03, 0·03	−0·03	–0·07, 0·001	–		–	
Lived in a refugee camp prior to arrival (Ref: No)								
Yes	−0·16	–0·33, 0·01	0·22	0·02, 0·43*	−0·17	–0·38, 0·03	0·27	0·005, 0·54*
Region of origin (Ref: Africa)								
Middle East	−0·24	–0·42, –0·04*	−0·07	–0·29, 0·16	−0·18	–0·41, 0·05	0·06	–0·24, 0·37
Asia	−0·01	–0·18, 0·16	−0·04	–0·25, 0·17	−0·12	–0·30, 0·07	−0·03	–0·27, 0·22

Each column is one linear regression model with all covariates.

*p < 0.05.

^a^Constrained pilesort where respondents were asked to group cards into parent, kid, both or do not belong piles titled as ‘Generational Differences’.

^b^Unconstrained pilesort where respondents were asked to group cards into whatever piles made sense to them titled as ‘General Piles’.

We also evaluated these competence measures with a subsample of one adolescent per household (*n* 38). In grouping foods in the unconstrained exercise, the association with having lived in a camp was the same in the constrained exercise. Adolescents who had lived in a refugee camp prior to migration exhibited more agreement to the overall responses (0·27 (0·005, 0·54).

## Discussion

Research examining the meaning of foods emphasises shared norms and beliefs about food and ways of eating within groups. We expanded this research by examining refugee adolescents’ cultural models of food in their new environment of resettlement, hypothesising that adolescents from diverse backgrounds and experiences would nonetheless exhibit shared cultural models when they resettled in a shared environment.

We evaluated what foods and drinks adolescents stopped and started consuming after migration. Many reported eating new foods and drinks (61 %) and no longer consuming others (58 %). New food and drinks included both healthy (apples and oranges) and potentially unhealthy items (pizza). Foods frequently listed as no longer eaten included rice and beans but also some items that are typically perceived and named by adolescents as ‘American’, including pizza, soda and chocolate milk. Dietary acculturation does not necessitate a wholesale change towards unhealthy foods^(36,37)^. Alternatively, no longer eating certain foods after migration can possibly be attributed to a lack of resources for acquiring these foods, or a desire to protect one’s ethnic identity through preserving foodways.

Using methods from cognitive anthropology, we found that adolescents who had recently moved to a city in the Southeastern USA on a refugee visa shared a cultural model about which foods are grouped together, including fruits, meal items, snacks and drinks. There was agreement across gender, age and number of siblings. Children in the same household had similar shared cultural models within the larger group. Children who had lived in a refugee camp were more likely to identify the general piles of items to be like the shared cultural model. These results are consistent with previous findings that shared environments lead young people to converge on similar understandings about foods^(18,38)^.

Adolescents showed consensus in grouping items in general and in specifying generational food differences. The cultural model of eating practices was shared across age, gender and number of siblings. Adolescents had all been in the USA for less than a year and come from diverse settings, yet they already exhibit a shared understanding of how foods relate to one another. Specifically, adolescents mention distinct groups or typologies of foods or identify foods consumed differently across generational strata.

Adolescents who had lived in a refugee camp were different in how they grouped items together in general (unconstrained) pilesort. They were more likely to group items like the overall shared model for the unconstrained pilesort compared with adolescents who had not lived in a refugee camp. It is possible that the allocation of food supplies to families in camps would make general item grouping applicable to the adolescents’ experiences regardless of camp status. Our framing suggests that competence in this sense simply refers to how much an individual agrees with a norm established by a group. In this case, the norm is established by a group of adolescents with varied backgrounds who agree on how food and beverage items relate to one another.

Our study provides preliminary insight into the processes of dietary acculturation among refugee adolescents during the first months of living in the USA. Adolescents in our sample share cultural models about generational preferences of foods. The extent to which these beliefs drive dietary choices among parents and adolescents was not examined. Our finding that adolescents who recently arrived in the USA generally agree about how foods relate to one another holds promise for nutrition and dietary interventions that engage with diverse adolescent groups. Researchers may glean more nuanced understanding of what drives dietary beliefs through qualitative or ethnographic research with adolescents and their caregivers.

Few studies have evaluated health behaviours post-migration among adolescent refugees. One study among immigrant and refugee adolescents (ages 12–17) in a school in North Carolina evaluated participants from thirty-five countries who had lived in the USA for less than a year^(39,40)^. The study determined that fruit, milk, fruit juice, soda and meat consumption increased post-arrival^(39)^. Furthermore, camp experience, sex, ethnicity and BMI were associated with these dietary changes^(40)^. That study did not differentiate between immigrant and refugee adolescents and focused on reported consumption *v*. the cultural models of food selection pre- and post-arrival. Pilesort methods have been utilised previosly, however, not with refugee adolescents. One pilesort study among low-income women in Santos, Brazil, asked about food categorisation^(41)^. Among ninety women, six clusters arose: home meals, convenience foods, special meals, fish, breads and cereals, and hot dogs. Rationales for food categories included frequency of consumption, degree of healthfulness, personal taste and meals in which the food was included ^(42)^. Among urban and rural caregivers in Sri Lanka, food classification highlighted a ‘hot’ and cold” paradigm reflecting Ayurvedic medical belief system^(42)^.

### Limitations and context

There are several limitations to this study. Unexplored factors, including food security, availability, accessibility, agency and intrahousehold dynamics, shape decisions around diet. For this study, we focus on beliefs surrounding food as a window into food preferences. The nature of our sampling and community partnership means all students attend the same school. This may entail that the cultural models of food are established in the school environment and thus do not develop individually as part of resettlement. Furthermore, some critics have argued that formal cognitive models of culture are limited and that cut-offs for what counts as ‘consensus’ are arbitrary^(43)^. The cards we developed did not fully encompass all items listed by our freelist exercise. There may be less consensus around certain items than others because they will be more familiar to youths from some places of origin than for others. Due to the sample size, our country-of-origin categories are very broad and encompass large regions of the world. Participants included in these categories had diverse and unique experiences that we are not able to disentangle. We have no information on participant’s ethnic identity within their country of origin as well as any religious affiliation, as these were items our community partners requested we not discuss with the youths. Ethnic identity and religious affiliation could entail cultural differences that may have implications for our results. For example, Afghanistan has at least fourteen major ethnolinguistic groups that differ in terms of diet, among other factors.

### Conclusions

Our study expands the applications of cognitive theories of culture to illuminate processes of dietary acculturation in the USA among adolescent refugees within 1 year of arrival. It demonstrates that foods commonly consumed change within months of resettlement. Based on our findings of a shared cultural model, nutrition programming efforts with adolescents may be generalisable across refugee origin groups. Our work also offers insights into how adolescents categorise the food landscape into adult foods and children’s foods. Adolescent’s classifications indicates that many healthy food options are not considered to be in the domains of children and teens.

## Supporting information

Jones-Antwi et al. supplementary material Jones-Antwi et al. supplementary material

## Supplementary material

For supplementary material accompanying this paper visit https://doi.org/10.1017/S1368980024002544
