# Supplementary material for: Sorting it out: perceptions of foods among newly arrived adolescent refugees in the Southeastern USA
Source: Public Health Nutr. 2024 Dec 26;28(1):e11. doi: 10.1017/S1368980024002544 (PMC11822612; doi:10.1017/S1368980024002544)
Supplement: Jones-Antwi et al. supplementary material [file S1368980024002544sup001.pdf]

APPENDIX

Figure A.1. Cards Used in Pilesort

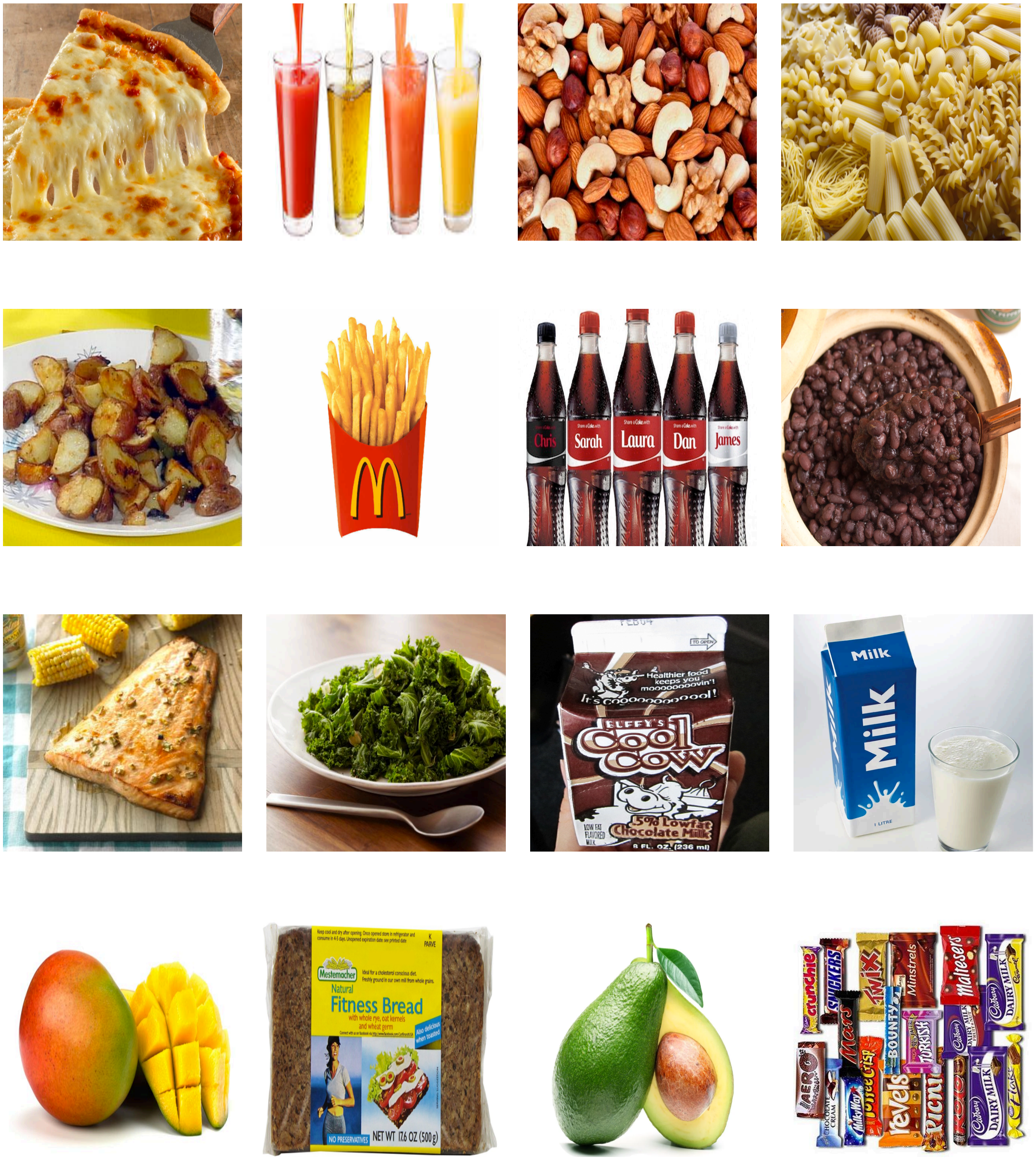

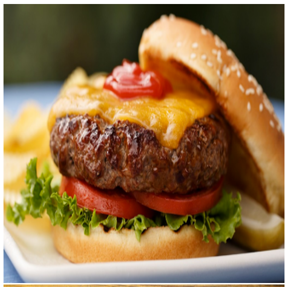

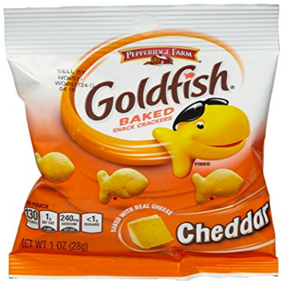
Pepperidge Farm
Goldfish
Baked Snack Crackers
Cheddar
NET WT 1 OZ (28g)

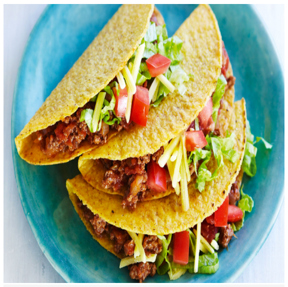

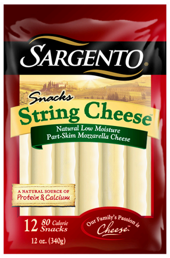
Sargento
Snacks
String Cheese
Natural Low Moisture Part-Skim Mozzarella Cheese
12 80 Calorie Snacks
12 oz. (340g)

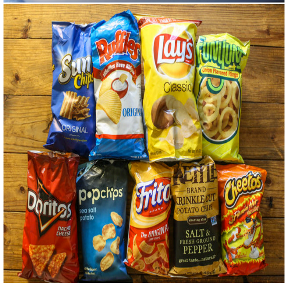
Ruffles
Lay's
Classic
Doritos
popchips
Fritos
Cheetos
Salt & Fresh Ground Pepper

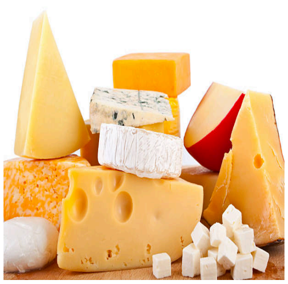

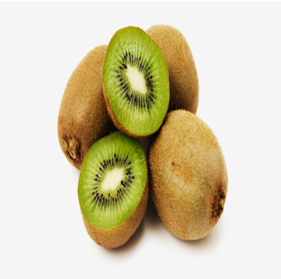

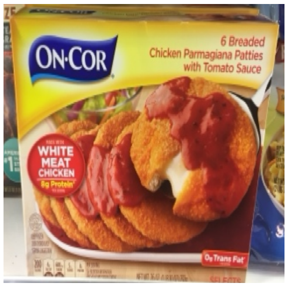
On-Cor
6 Breaded Chicken Parmagiana Patties with Tomato Sauce
White Meat Chicken

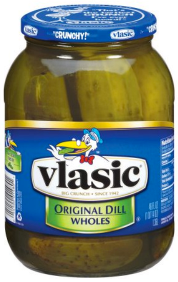
vlasic
Original Dill Wholes

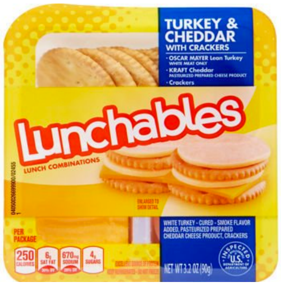
Turkey & Cheddar with Crackers
Lunchables
Lunch Combinations

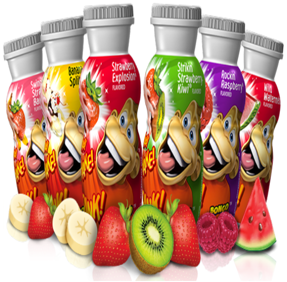

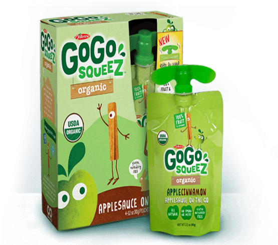
GoGo squeeZ
organic
Applesauce

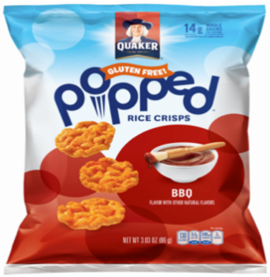
Quaker
Gluten Free
popped
Rice Crisps
BBQ

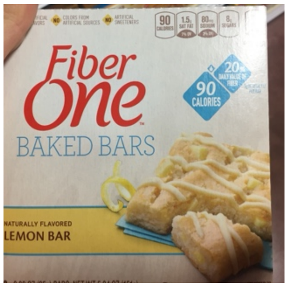
Fiber One
90 Calories
Baked Bars
Naturally Flavored
Lemon Bar

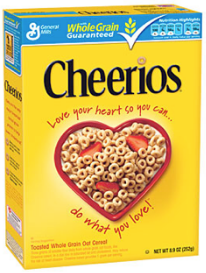
Whole Grain Guaranteed
Cheerios
Love your heart so you can...
do what you love!

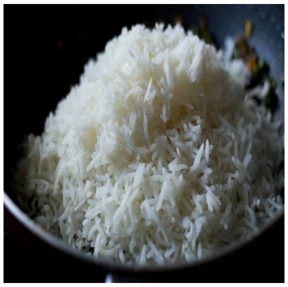

Figure A.2. Multidimensional scaling plot (stress = 0.211) and cluster analysis for one adolescent per household (n=38) unconstrained pilesorts of 30 foods and beverages

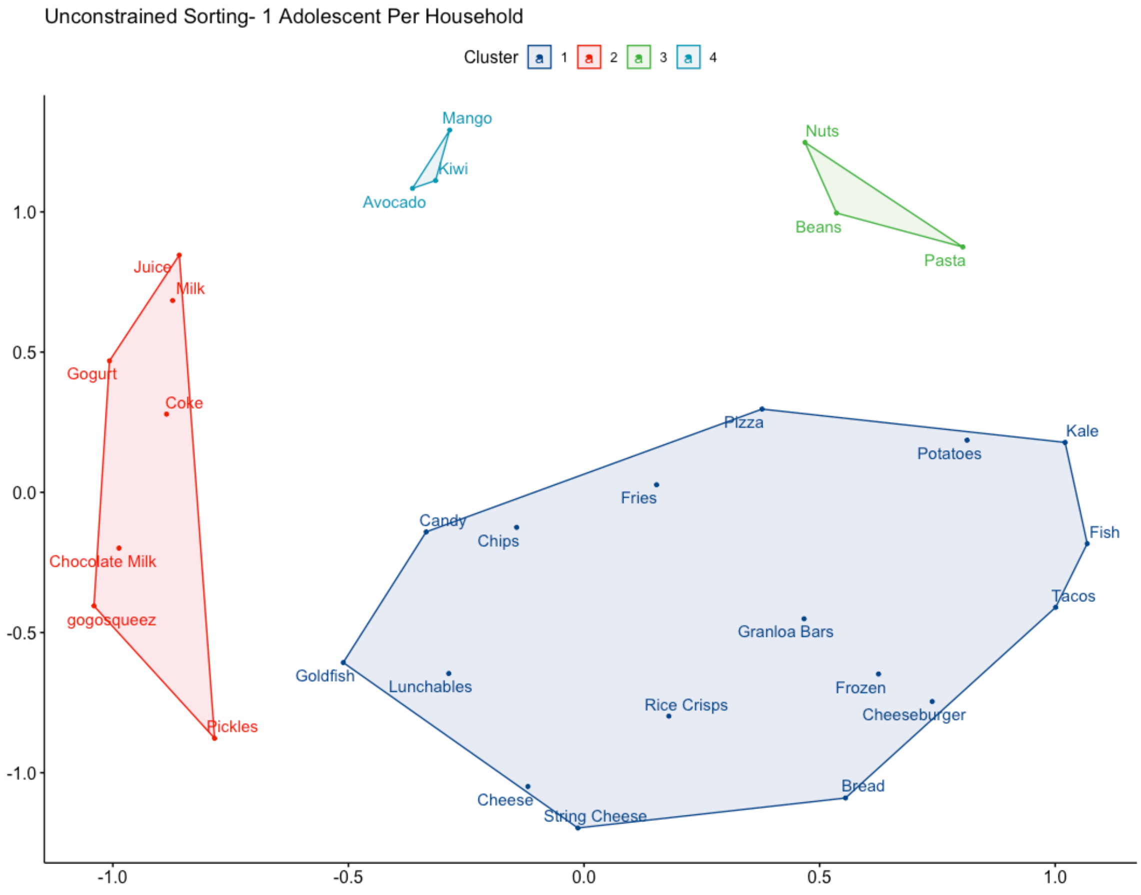

Note:
1: American Foods; 2: Snacks and Drinks; 3: Dense Foods; 4: Fruits

Figure A.3. Multidimensional scaling plot (stress = 0.224) and cluster analysis for one adolescent per household (n=38) constrained pilesorts of 30 foods and beverages

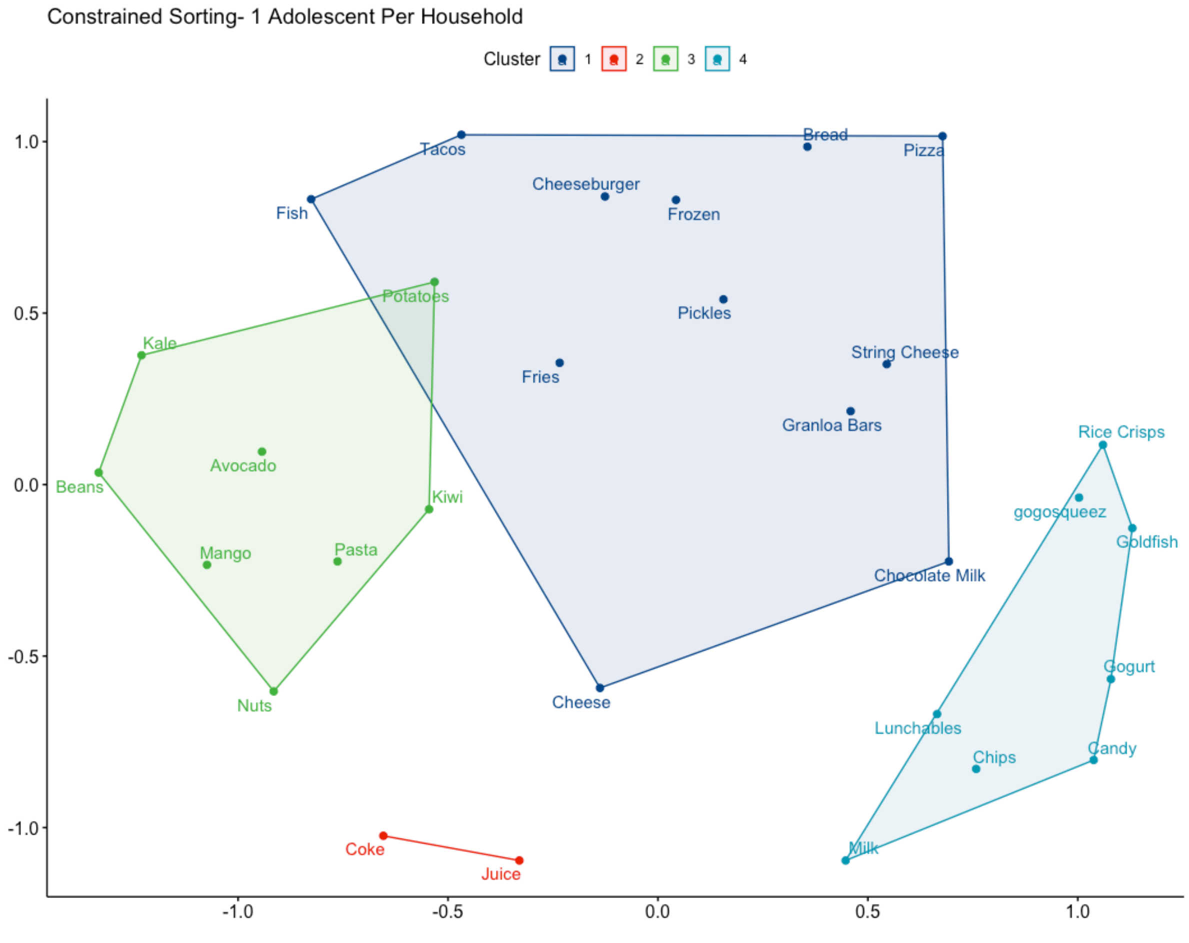

Note:
Legend:

1: Both; 2: Don't Belong; 3: Parent Foods and Drinks; 4: Adolescent Foods and Drinks
